# Supplementary material for: MiLoPYP: self-supervised molecular pattern mining and particle localization in situ
Source: Nat Methods. 2024 Sep 9;21(10):1863–72. doi: 10.1038/s41592-024-02403-6 (PMC11468773; doi:10.1038/s41592-024-02403-6)
Supplement: Supplementary file 1 — Supplementary Fig. 1 and Tables 1–3. [file 41592_2024_2403_MOESM1_ESM.pdf]

nature methods

Supplementary information

https://doi.org/10.1038/s41592-024-02403-6

MiLoPYP: self-supervised molecular pattern mining and particle localization in situ

In the format provided by the authors and unedited

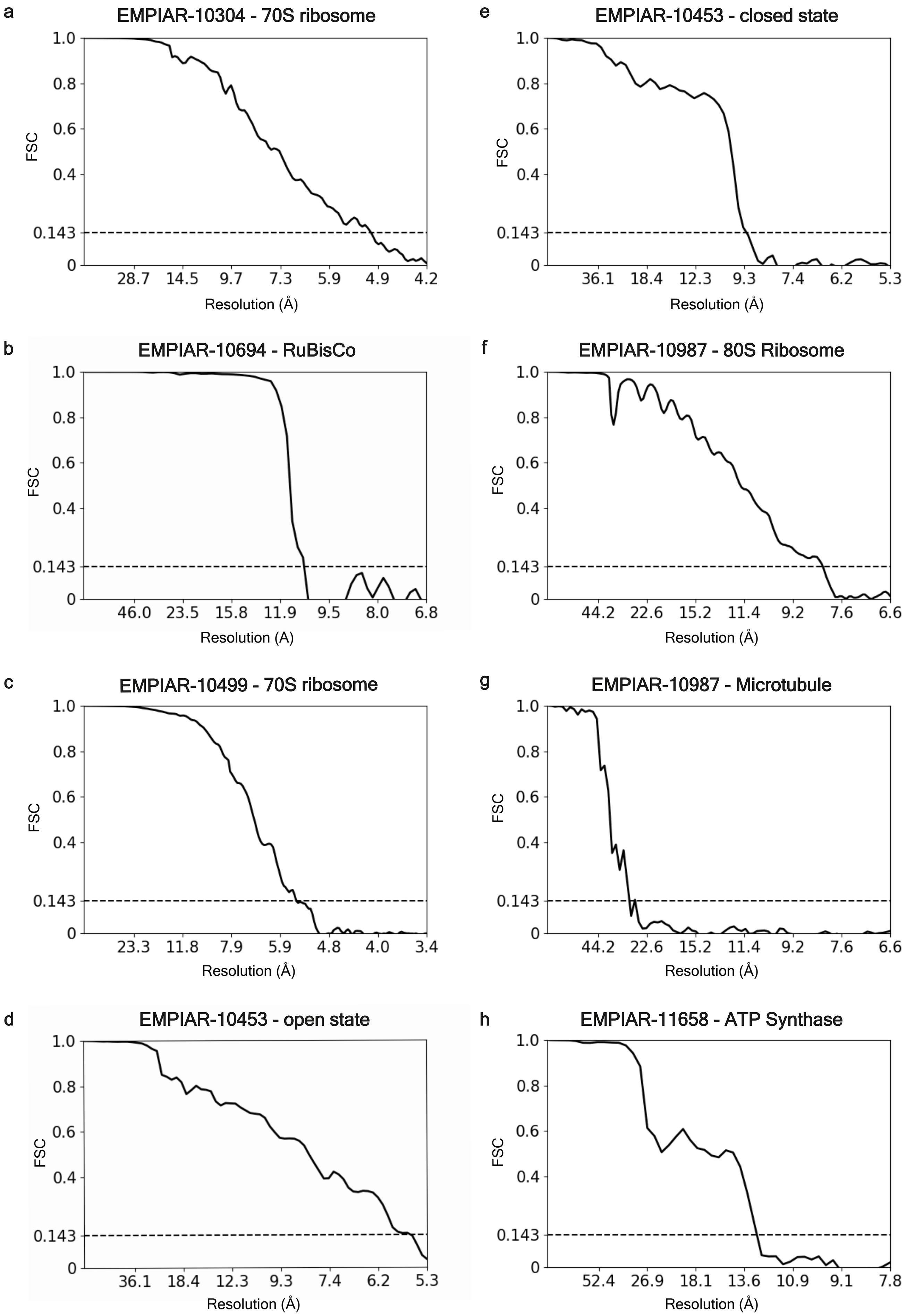

Supplementary Figure 1: Fourier Shell Correlation (FSC) plots between half-maps for all structures determined using particles picked by MiLoPYP. a) Purified 70S ribosome from EMPIAR-10304 (5.0 Å). **b)** In-situ RuBisCo from EMPIAR-10694 (11.0 Å). **c)** In-situ 70S ribosome from EMPIAR-10499 (5.4 Å). **d-e)** Membrane-bound Sars-CoV-2 spikes from EMPIAR-10453 in open (d) and closed (e) states (5.6 Å and 9.3 Å, respectively). **f-g)** 80S ribosome (f) and microtubule (g) structures obtained from in-situ lamellae available from EMPIAR-10987 (8.2 Å and 27.4 Å, respectively). **h)** In-situ ATP Synthase from EMPIAR-11658 (13.0 Å). All resolutions were determined using the 0.143-cutoff of the FSC between half-maps.

Dataset		EMPIAR-10304	EMPIAR-10987	EMPIAR-10453	SHREC-21
**Number of tomograms**		2	2	2	1
**F1 Scores**					
	DeepFinder	0.22	0.04	N/A	**0.83**
	DeePiCt	0.29	0.33	N/A	N/A
	TomoTwin (reference-based)	0.74	0.64	0.31	0.57
	MiLoPYP	**0.81**	**0.79**	**0.61**	0.58

Supplementary Table 1: Performance comparison between MiLoPYP and other deep learning-based approaches. We present F1 scores for DeepFinder, DeePiCt, TomoTwin, and MiLoPYP on tomograms from EMPIAR-10304, EMPIAR-10987, EMPIAR-10453 and SHREC 2021 (average scores over all classes are reported in this case). The fully-supervised approach DeepFinder performs best on the synthetic SHREC 2021 dataset, while MiLoPYP outperforms all other approaches on the EMPIAR datasets.

Dataset		EMPIAR-10304	EMPIAR-10453	EMPIAR-10499	EMPIAR-10987
**Number of tomograms**		2	2	2	1
**Without refinement module**					
	Precision	0.58	0.15	0.34	0.42
	Recall	0.64	0.84	0.63	0.67
	F1	0.61	0.25	0.44	0.52
**With refinement module**					
	Precision	0.83	0.54	0.72	0.76
	Recall	0.86	0.69	0.76	0.85
	F1	0.84	0.61	0.74	0.8

Supplementary Table 2: Contribution of the contrastive learning module on particle picking accuracy. We present Precision, Recall, and F1 scores in the absence and presence of the refinement module when running MiLoPYP on four EMPIAR datasets, showing a significant improvement in picking accuracy when adding the refinement module.

Dataset (EMPIAR ID)	10304	10499	10453	10987		11658	10694
**Downsampled Data size**							
Tomogram size (x,y,z)	512x512 x256	512x512 x256	512x512 x256	512x512 x256		512x512 x256	1024x1024 x256
Tilt series size (width, height, tilts)	512x512 x41	512x512 x41	512x512 x41	512x512 x41		512x512 x41	1024x1024 x65
Number of tomograms	11	65	266	21		48	1
**Cellular content exploration time (hh:mm:ss)**							
Guassian filtering (per tomo)	0:00:02.5	0:00:02.5	0:00:02.5	0:00:02.5		0:00:02.5	0:00:06.10
Difference of Gaussians (per tomo)	0:00:10.3	0:00:10.3	0:00:10.3	0:00:10.3		0:00:10.3	0:00:14.5
Number of tomograms for training	3	5	3	3		3	1
Number of subvolumes	45,460	32,174	38,750	18,239		27,171	55,986
Training (300 epochs)	1:40:32	0:55:48	1:10:32	0:21:30		0:41:21	2:30:41
Overclustering	0:01:07	0:00:48	0:00:57	0:00:21		0:00:28	0:01:42
Total exploration time	1:41:39	0:56:36	1:11:29	0:21:51		0:41:49	2:32:23
**Few shot particle localization(hh:mm:ss)**	**Ribosome**	**Ribosome**	**Spike**	**Ribosome**	**Microtubule**	**ATP Synthase**	**RuBisCo**
Number of particles for training	230	195	144	137	102	247	86
Training (10 epochs)	0:06:48	0:03:41	0:03:25	0:02:48	0:02:21	0:08:17	0:02:10
Inference	0:00:18	0:01:37	0:03:25	0:00:38	0:00:31	0:01:13	0:00:02
Microtubule post-processing	N/A	N/A	N/A	N/A	0:00:15	N/A	N/A
Total particle-picking time	0:07:06	0:05:18	0:06:50	0:03:26	0:03:07	0:09:30	0:02:12
Total picked particles	8,965	23,285	23,388	6,068	1,761	4,105	36,345
Used particles for 3D reconstruction	6,051	17,381	Open 9,194 Closed 3,740	4,570	1,761	2,577	35,352
Resolution	5.0 Å	5.4Å	5.6 Å 9.3 Å	8.6 Å	37.0 Å	13.0 Å	11.0 Å

Supplementary Table 3: Data processing parameters and timing statistics for cellular content exploration and particle localization modules in MiLoPYP. Data is presented for tilt-series from in-vitro ribosomes (EMPIAR-10304), in-situ ribosomes (EMPIAR-10499), native Sars-CoV-2 spikes (EMPIAR-10453), in-situ lamellae (EMPIAR-10987), *S. cerevisiae* cells (EMPIAR-11658), and *Chlamydomonas reinhardtii* cells (EMPIAR-10694). Processing times are reported on a single NVIDIA V100 GPU with 32GB RAM.
