# Supplementary material for: MiLoPYP: self-supervised molecular pattern mining and particle localization in situ
Source: Nat Methods. 2024 Sep 9;21(10):1863–72. doi: 10.1038/s41592-024-02403-6 (PMC11468773; doi:10.1038/s41592-024-02403-6)
Supplement: Supplementary file 2 — Reporting Summary [file 41592_2024_2403_MOESM2_ESM.pdf]

nature portfolio

Corresponding author(s): Alberto Bartesaghi

Last updated by author(s): Jul 10, 2024

Reporting Summary

Nature Portfolio wishes to improve the reproducibility of the work that we publish. This form provides structure for consistency and transparency in reporting. For further information on Nature Portfolio policies, see our Editorial Policies and the Editorial Policy Checklist.

Statistics

For all statistical analyses, confirm that the following items are present in the figure legend, table legend, main text, or Methods section.

n/a	Confirmed	
☐	☒	The exact sample size (*n*) for each experimental group/condition, given as a discrete number and unit of measurement
☐	☒	A statement on whether measurements were taken from distinct samples or whether the same sample was measured repeatedly
☒	☐	The statistical test(s) used AND whether they are one- or two-sided<br>*Only common tests should be described solely by name; describe more complex techniques in the Methods section.*
☒	☐	A description of all covariates tested
☒	☐	A description of any assumptions or corrections, such as tests of normality and adjustment for multiple comparisons
☐	☒	A full description of the statistical parameters including central tendency (e.g. means) or other basic estimates (e.g. regression coefficient) AND variation (e.g. standard deviation) or associated estimates of uncertainty (e.g. confidence intervals)
☒	☐	For null hypothesis testing, the test statistic (e.g. *F*, *t*, *r*) with confidence intervals, effect sizes, degrees of freedom and *P* value noted<br>*Give P values as exact values whenever suitable.*
☒	☐	For Bayesian analysis, information on the choice of priors and Markov chain Monte Carlo settings
☐	☒	For hierarchical and complex designs, identification of the appropriate level for tests and full reporting of outcomes
☒	☐	Estimates of effect sizes (e.g. Cohen's *d*, Pearson's *r*), indicating how they were calculated

Our web collection on statistics for biologists contains articles on many of the points above.

Software and code

Policy information about availability of computer code

Data collection	No new data collection was performed for this study.
Data analysis	Publicly available software used for data analysis: nextPYP v0.6.5 (http://nextpyp.app), PyTorch v1.11.0, umap v0.5.3, and Phoenix v0.0.27.<br>Custom software developed for this study: MiLoPYP v0.6.5 (https://github.com/nextpyp/cet_pick).

For manuscripts utilizing custom algorithms or software that are central to the research but not yet described in published literature, software must be made available to editors and reviewers. We strongly encourage code deposition in a community repository (e.g. GitHub). See the Nature Portfolio guidelines for submitting code & software for further information.

Data

Policy information about availability of data

All manuscripts must include a data availability statement. This statement should provide the following information, where applicable:

- Accession codes, unique identifiers, or web links for publicly available datasets
- A description of any restrictions on data availability
- For clinical datasets or third party data, please ensure that the statement adheres to our policy

This study utilized raw tilt-series available from the Electron Microscopy Public Image Archive (EMPIAR) database under accession numbers 10304, 10499, 10453, 10987, and 11658 and cryo-EM maps available from the Electron Microscopy Data Bank (EMDB) under accession numbers 23358, 11650, 11347, 10061 and 33118. Cryo-EM density maps produced in this study were deposited in the EMDB under accession nos. EMD-45261 for EMPIAR-10304, EMD-45266 for EMPIAR-10499,

EMD-45267 and EMD-45268 for EMPIAR-10453, EMD-45269 for EMPIAR-10694, EMD-45270 and EMD-45271 for EMPIAR-10987, and EMD-45272 for EMPIAR-11658.

Human research participants

Policy information about studies involving human research participants and Sex and Gender in Research.

Reporting on sex and gender	No Human research participants were used in this study.
Population characteristics	N/A
Recruitment	N/A
Ethics oversight	N/A

Note that full information on the approval of the study protocol must also be provided in the manuscript.

Field-specific reporting

Please select the one below that is the best fit for your research. If you are not sure, read the appropriate sections before making your selection.

☒ Life sciences ☐ Behavioural & social sciences ☐ Ecological, evolutionary & environmental sciences

For a reference copy of the document with all sections, see nature.com/documents/nr-reporting-summary-flat.pdf

Life sciences study design

All studies must disclose on these points even when the disclosure is negative.

Sample size	All raw data used in this study were obtained from datasets deposited in the EMPIAR database. The total number of tilt-series available for each entry was used, as follows: EMPIAR-10304 (12 tilt-series), EMPIAR-10499 (65 tilt-series), EMPIAR-10453 (266 tilt-series), EMPIAR-10987 (20 tilt-series), and EMPIAR-11658 (260 tilt-series). Datasets were selected to represent a wide variety of in vitro and in situ cryo-ET samples, as needed to fully evaluate the performance of MiLoPYP.
Data exclusions	All data from the corresponding entries were used for processing. No data were excluded from the analyses.
Replication	Detailed protocols for obtaining the final maps were documented and used by at least three different lab members to reproduce the results. Replication attempts resulted in similar embeddings, same number of particles, and maps with identical resolutions.
Randomization	Randomization was not relevant in this study. All tilt-series were used for the analyses so no randomization was needed.
Blinding	Blinding was not relevant for this study because all tilt-series were used for the analyses. No data were left out.

Reporting for specific materials, systems and methods

We require information from authors about some types of materials, experimental systems and methods used in many studies. Here, indicate whether each material, system or method listed is relevant to your study. If you are not sure if a list item applies to your research, read the appropriate section before selecting a response.

Materials & experimental systems

n/a	Involved in the study
☒	☐ Antibodies
☒	☐ Eukaryotic cell lines
☒	☐ Palaeontology and archaeology
☒	☐ Animals and other organisms
☒	☐ Clinical data
☒	☐ Dual use research of concern

Methods

n/a	Involved in the study
☒	☐ ChIP-seq
☒	☐ Flow cytometry
☒	☐ MRI-based neuroimaging
